# Supplementary material for: Soil Metabolomics Predict Microbial Taxa as Biomarkers of Moisture Status in Soils from a Tidal Wetland
Source: Microorganisms. 2022 Aug 16;10(8):1653. doi: 10.3390/microorganisms10081653 (PMC9416152; doi:10.3390/microorganisms10081653)
Supplement: Supplementary file 1 [file microorganisms-10-01653-s001.zip › microorganisms-1714603-supplementary.pptx]

## Slide 1
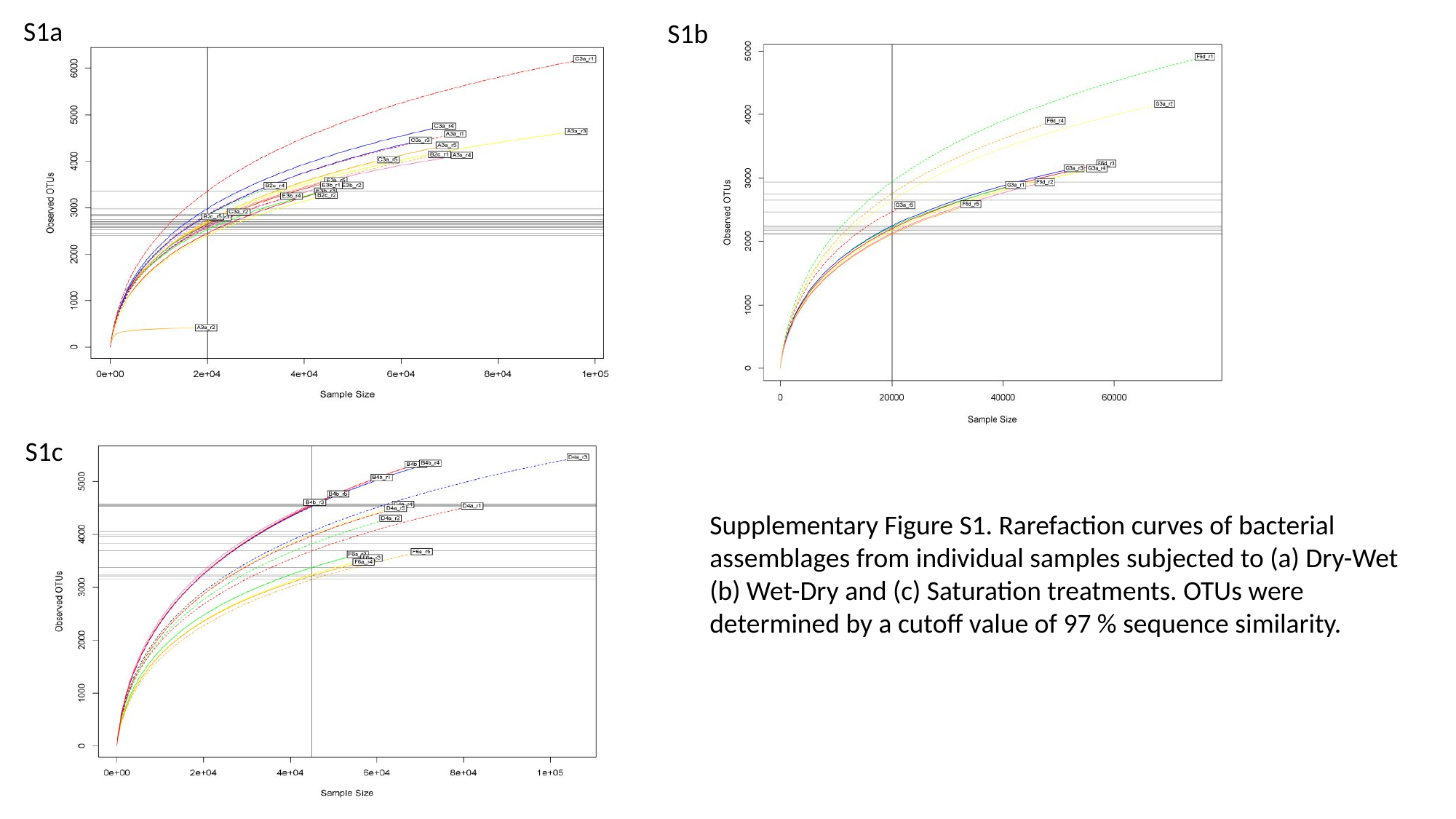

S1a
S1b
S1c
Supplementary Figure S1. Rarefaction curves of bacterial assemblages from individual samples subjected to (a) Dry-Wet (b) Wet-Dry and (c) Saturation treatments. OTUs were determined by a cutoff value of 97 % sequence similarity.

## Slide 2
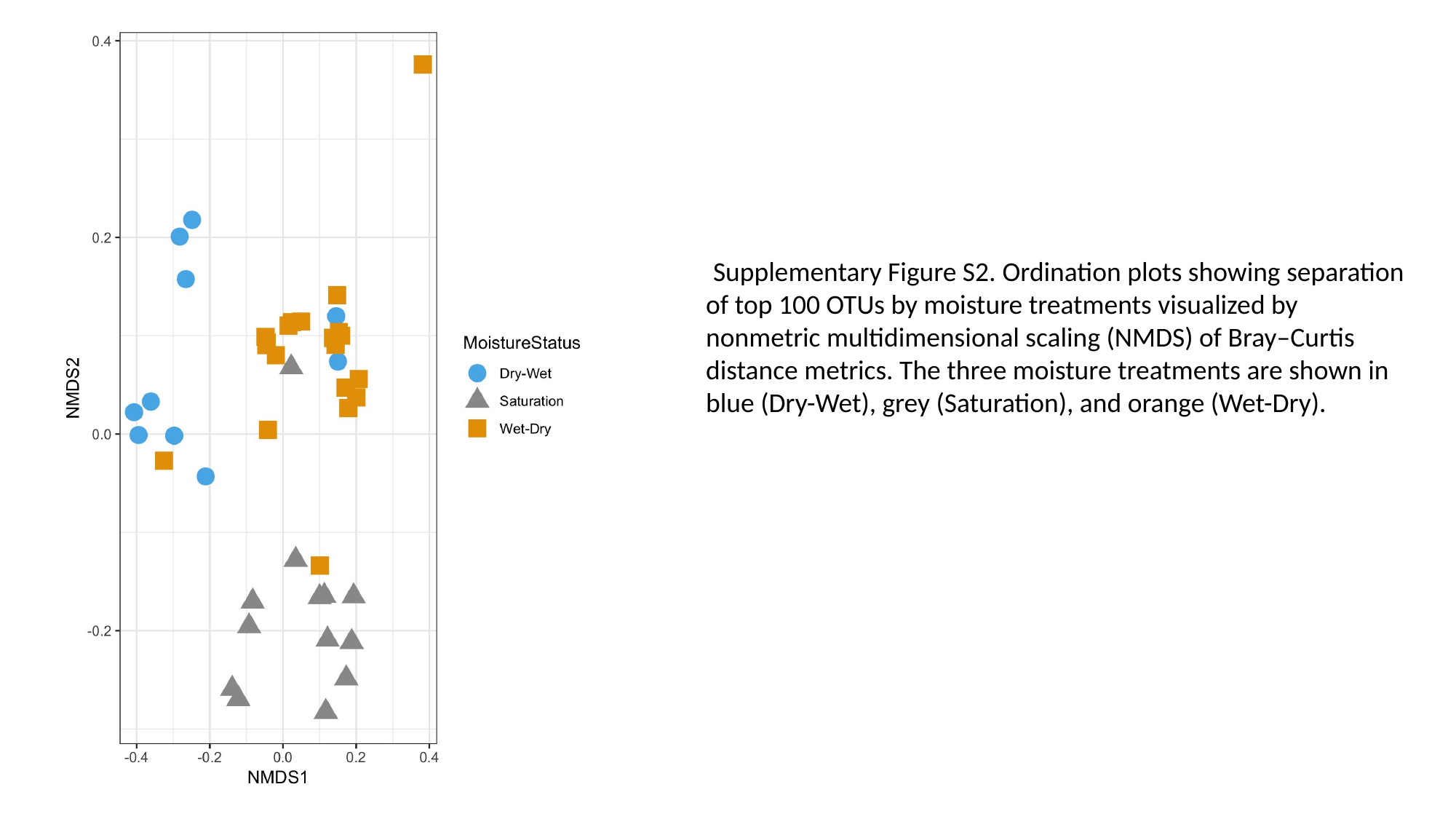

Supplementary Figure S2. Ordination plots showing separation of top 100 OTUs by moisture treatments visualized by nonmetric multidimensional scaling (NMDS) of Bray–Curtis distance metrics. The three moisture treatments are shown in blue (Dry-Wet), grey (Saturation), and orange (Wet-Dry).

## Slide 3
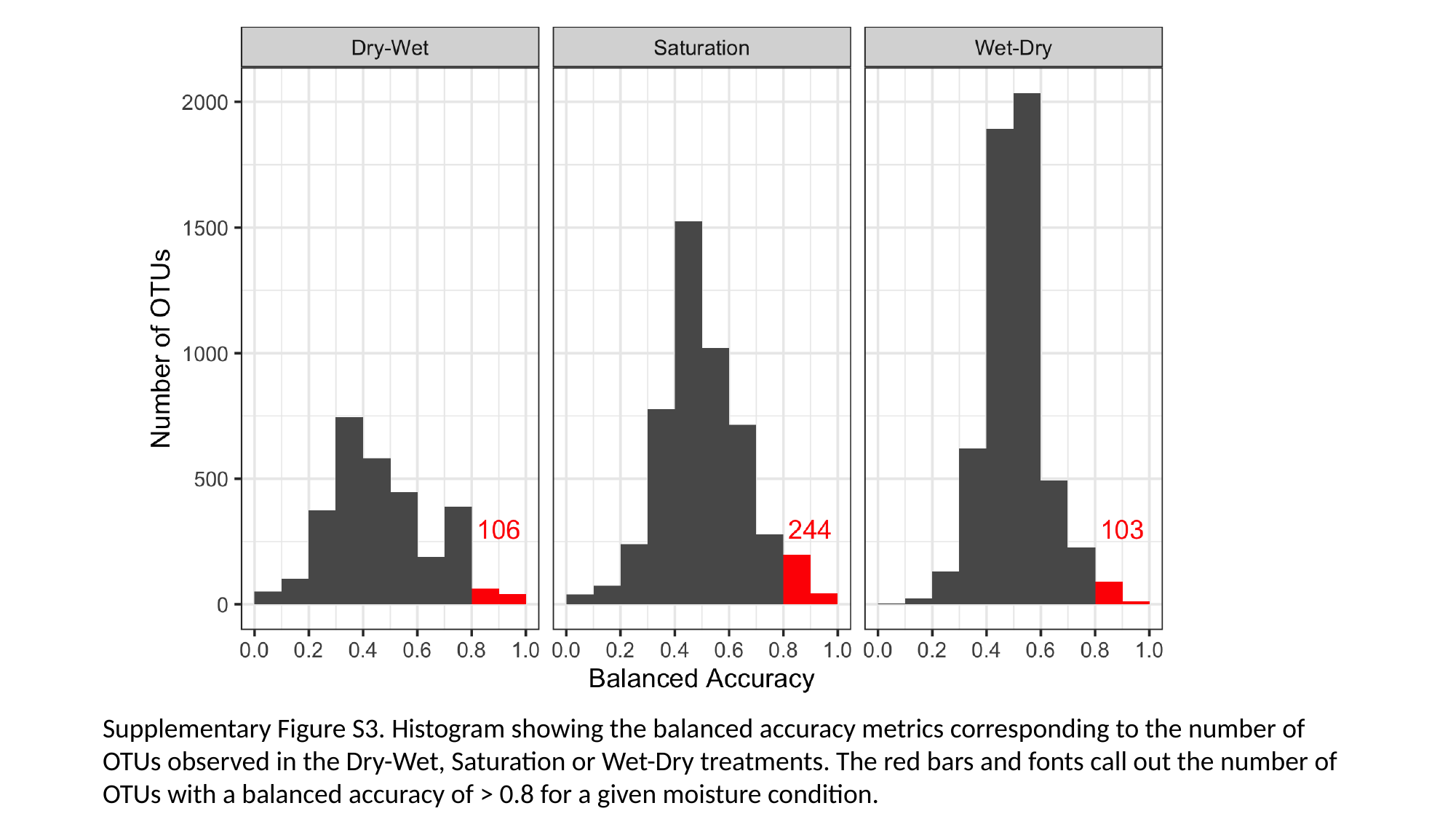

Supplementary Figure S3. Histogram showing the balanced accuracy metrics corresponding to the number of OTUs observed in the Dry-Wet, Saturation or Wet-Dry treatments. The red bars and fonts call out the number of OTUs with a balanced accuracy of > 0.8 for a given moisture condition.

## Slide 4
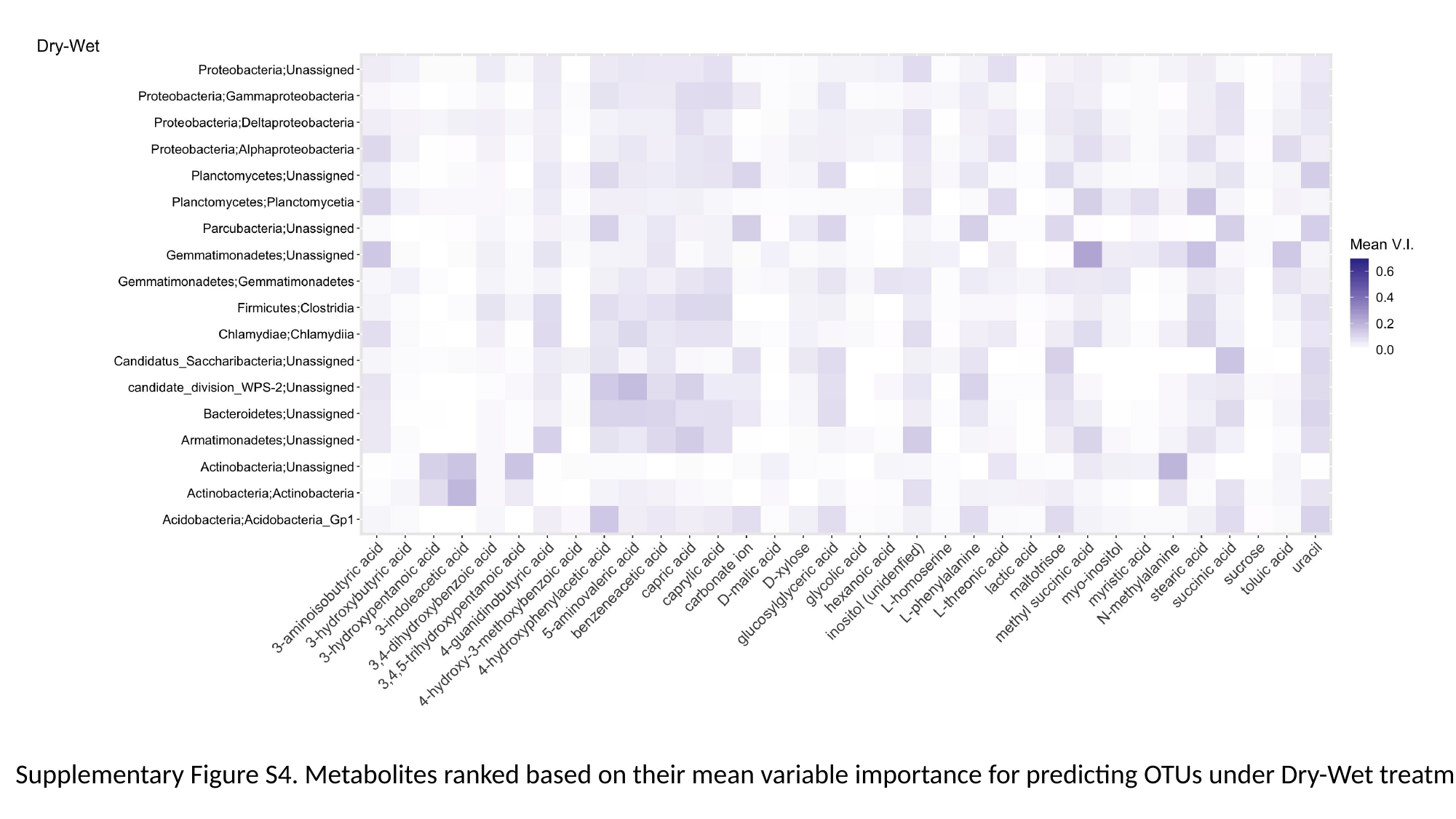

Supplementary Figure S4. Metabolites ranked based on their mean variable importance for predicting OTUs under Dry-Wet treatment.
